# Sensitivity and Specificity of the ADOS-2 Algorithm in a Large German Sample

**DOI:** 10.1007/s10803-018-3750-3

**Published:** 2018-09-20

**Authors:** Juliane E. Medda, Hannah Cholemkery, Christine M. Freitag

**Affiliations:** Department of Child and Adolescent Psychiatry, Psychosomatics and Psychotherapy, Autism Research and Intervention Centre of Excellence, University Hospital Frankfurt, Goethe University, Frankfurt am Main, Germany

**Keywords:** Psychometric assessment, Child psychiatric disorder, Autism spectrum disorder, Diagnostic validity

## Abstract

The aim of the present study was to establish diagnostic validity of the new algorithm of the Autism Diagnostic Observation Scale, the ADOS-2, to differentiate between ASD and other clinically relevant psychiatric and developmental disorders in a large German sample. Validity of ADOS and ADOS-2 diagnostic algorithms was established in 826 individuals (*n* = 455 autism, *n* = 216 autism spectrum, *n* = 155 non-ASD patients) by receiver operating curves. Confidence intervals overlapped largely for ADOS and ADOS-2 algorithms, confirming diagnostic validity of both algorithms. Adding information of the Social Communication Questionnaire and the Social Responsiveness Scale resulted in slightly improved classification rates for autism in Module 4. We thus replicated previous findings of the diagnostic validity of the ADOS-2 algorithms.

Autism spectrum disorders (ASD) are neurodevelopmental disorders characterized by impaired social communication and restricted and repetitive behavior (American Psychiatric Association 2013). The Autism Diagnostic Observation Schedule (ADOS; Lord et al. 2000) in combination with the Autism Diagnostic Interview-revised (ADI-R; Rutter et al. 2003, b) are considered gold standard for diagnosing autism according to ICD-10 and DSM-IV-TR (Falkmer et al. 2013; Ozonoff et al. 2005). In addition to an autism cut-off, the ADOS also provides an autism spectrum cut-off, which supports a diagnosis of Asperger Syndrome, atypical autism, or pervasive developmental disorder-not otherwise specified (PDD-NOS) according to ICD-10 (World Health Organization 1992) or DSM-IV-TR (American Psychiatric Association 2000). In the current DSM-5, these disorders are subsumed under the new diagnostic category Autism Spectrum Disorder (American Psychiatric Association 2013; Freitag 2014).

The ADOS is a direct observation schedule and consists of four modules (Lord et al. 2000). Module specific items are combined into a diagnostic algorithm with module specific cut-off scores for autism and autism spectrum versus non-ASD. Psychometric data provided by the authors (Lord et al. 2000) showed a high sensitivity between 86 and 100% and a moderate to high specificity between 68 and 100% to differentiate autism and non-ASD as well as autism spectrum and non-ASD. Subsequent studies found an association of chronological age, cognitive and verbal functioning on different algorithm scores (Bildt et al. 2004; Bishop and Norbury 2002; Gotham et al. 2007; Joseph et al. 2002; Lord et al. 2000), implying that the ADOS does not measure equally across heterogeneous ASD populations. In addition, only social interaction and communication items are part of the original ADOS algorithm, despite the standardized assessment of play, restricted, repetitive behaviors and interests during observation. To address these issues a new algorithm (ADOS-2) for Modules 1–4 was developed (Gotham et al. 2007, 2008; Hus and Lord 2014). Gotham et al. established the new algorithm in a clinical sample to differentiate autism and autism spectrum (not including autism) from individuals with developmental or language delay or other psychiatric disorders (Gotham et al. 2007). The sample was divided by age and language level into homogeneous cells with minimal correlations of ADOS total scores with age, verbal IQ and verbal mental age. Item distribution within each cell was then studied to select the best differentiating items between autism, autism spectrum and non-ASD diagnoses. The diagnostic algorithm of the ADOS-2 was derived from the Social Affect (SA) and Restrictive, Repetitive Behavior (RRB) domains by comparing its sensitivity and specificity to the original ADOS algorithm.

For Module 1, separate ADOS-2 algorithms for verbal and nonverbal individuals were established. For Module 2, separate ADOS-2 algorithms for children up to 5 years of age and children 5 years and older were defined. The new algorithms showed a higher specificity for autism in Module 1, nonverbal group, while sensitivity remained similar. In all other modules and subgroups sensitivity increased and specificity decreased for autism. For autism spectrum versus non-ASD, specificity increased slightly or stayed similar in all modules. Sensitivity stayed similar or increased slightly in all modules (Gotham et al. 2007).

In subsequent studies, sensitivity of Module 1–3 (diagnostic groups autism versus non-ASD and autism spectrum versus non-ASD) stayed similar or was slightly increased when comparing ADOS-2 to ADOS algorithms (Gotham et al. 2008; Bildt et al. 2009; Oosterling et al. 2010; Kamp-Becker et al. 2013). In one study, sensitivity decreased slightly for Module 2, younger than 5 years (Gotham et al. 2008).

Specificity stayed similar or increased nominally for all algorithms when using the ADOS-2 algorithm compared to the original algorithm in most studies (Lord et al. 2000; Gotham et al. 2008; Oosterling et al. 2010). Other authors found a nominal decrease in specificity when applying the new ADOS-2 algorithm for the autism versus non-ASD group (Bildt et al. 2009; Kamp-Becker et al. 2013), or the autism spectrum versus non-ASD group compared to the original ADOS algorithms (Kamp-Becker et al. 2013).

For Module 4, sensitivity stayed similar or increased slightly when comparing the original and ADOS-2 algorithm (Hus and Lord 2014; Pugliese et al. 2015; Bildt et al. 2016). Specificity increased in two studies (Hus and Lord 2014; Bildt et al. 2016). In another study, specificity decreased when applying the ADOS-2 algorithm (Pugliese et al. 2015).

The sample sizes of these studies were highly variable. Especially for Module 4, only a few studies - with inconsistent results - have been published to date. Given that sensitivity and specificity rarely exceeded 0.9 in all studies, the combination of ADOS-2 algorithm data with frequently used parent questionnaires, such as the Social Communication Questionnaire (SCQ; Rutter et al. 2003a, b) or the Social Responsiveness Scale (SRS; Constantino and Gruber 2005) may improve sensitivity and specificity.

With the aim of establishing establish cross-cultural diagnostic accuracy data for all 4 ADOS-2 modules in a clinically relevant sample, we compared sensitivity and specificity of ADOS and ADOS-2 algorithms in a large German sample of children, adolescents and adults with autism, autism spectrum as well as non-ASD developmental and psychiatric disorders. We expected to find similar sensitivity and specificity in Module 1 to Module 4 ADOS-2 algorithms as in the US-American samples, implementing the same cut-off values. Adding to previous studies, we also tested if correct diagnostic classification improved by adding parent information derived from the SRS and the SCQ to the most frequently obtained ADOS-2 Modules 3 and 4.

## Method

### Participants

ADOS algorithm data of *N* = 826 children, adolescents, and adults aged 2–40 years were re-evaluated using the revised ADOS-2 algorithm (Gotham et al. 2007, 2008; Hus and Lord 2014). Individuals were clinically or self-referred for suspected ASD to the Department of Child and Adolescent Psychiatry, Psychosomatics and Psychotherapy, University Hospital Frankfurt am Main. All patients with one or several psychiatric diagnoses or developmental disorder were included. Data were collected over a period of 16 years (1998–2014). Children, adolescents and adults carrying genetic syndromes, CNVs or mutations possibly underlying their psychiatric or developmental symptoms were included. We excluded from this study only individuals with a main diagnosis of a chronic medical or neurological disorder. Informed consent concerning the use of clinical data for further data analyses was obtained from parents and legal guardians or adult participants.

### Measures

The ADOS is a semi-structured assessment of social interaction, communication and play with the goal to provide situations that elicit spontaneous behaviors in standardized contexts (Lord et al. 2000). Assessment is possible with four different modules, each appropriate for different developmental and language levels (Lord et al. 2000). An experienced clinical psychologist or psychiatrist, who was trained to ADOS research standards, conducted the ADOS. Throughout the assessment, the examiner is coding items, which are grouped into the following domains: communication, social interaction, restricted and repetitive stereotyped behaviors and interests. In the original diagnostic algorithm, separate cut-offs for the communication and social domains as well as a combined cut-off must be met to receive an ADOS classification of autism or autism spectrum (Gotham et al. 2008; Lord and Rutter 2000). To receive an autism or autism spectrum diagnosis by the ADOS-2 algorithm an individual`s score must exceed a total cut-off score of the combined SA and RRB domain score (Lord and Rutter 2012; Gotham et al. 2007; Hus and Lord 2014). To facilitate comparability with previous research we used the same age ranges for the different modules as in the original publications: 12 years and younger for module 1 and 2 and 16 years and younger for Module 3 (Gotham et al. 2007). In accordance with the original publication for ADOS-2 (Hus and Lord 2014), we did not set a specific age range for Module 4.

The SCQ is a screening instrument assessing communication and social functioning and was designed as a questionnaire version of the ADI-R (Rutter et al. 2003a, b). We implemented the lifetime version in this study. The SCQ has 40 items with yes/no answers to be filled out by the primary caregiver. It can be used for individuals aged 4 years and older with a mental age of 2 years and older (Rutter et al. 2003a, b). The recommended cut-off value for autism is 15 (Rutter et al. 2003a, b). We thus used this cut-off value when comparing autism to non-ASD in the logistic regression. When comparing autism spectrum and autism patients against non-ASD patients in the logistic regression we chose a cut-off of 11. This cut-off has been recommended especially for high-functioning ASD because of increased sensitivity differentiating frequent child psychiatric disorders, such as attention-deficit/hyperactivity disorder (Kroger et al. 2011; Schwenck and Freitag 2014).

The SRS is a parent questionnaire assessing social responsiveness in 4- to 18-year-old children and adolescents (Constantino and Gruber 2005). 65 items can be answered on a 4-point Likert-Scale from 0 (not true) to 3 (almost always true), resulting in a maximum total score of 195. As recommended by the German clinical diagnostic guidelines for Autism Spectrum Disorders based on meta-analyses of SRS studies (AWMF 2016, awmf.org; Vllasaliu et al., submitted) a total score of ≥ 75 was chosen as cut-off in the logistic regression for all diagnostic groups. This cut-off has been shown to have balanced sensitivity and specificity (Bölte et al. 2011).

IQ was measured by standardized IQ tests with current German norms. IQ data were obtained for 74% of the patients. IQ could not be obtained due to either lack of compliance (especially severely affected young children) or cognitive testing at a different institution, which did not provide detailed data. Of the individuals with IQ data, 51.2% were tested with different German versions of the Wechsler Scales (HAWIK-IV/WISC-IV, Petermann and Petermann 2007; WPPSI-III, Petermann and Lipsius 2009; WAIS-III/WIE, Aster et al. 2006; HAWIK-III/WISC-III, Tewes et al. 1999). 42.1% were tested with nonverbal tests (Culture Fair Intelligence Test, Weiß 2006; Snijders–Oomen nonverbal Intelligence Test 2½–7, Tellegen et al. 2007; Standard Progressive Matrices, SPM, Raven 2009; Coloured Progressive Matrices, CPM, Raven et al. 2001) and 6.7% were tested with other tests (Peabody Picture Vocabulary Test, Dunn and Dunn 2007; K-ABC, Melchers and Preuß 2009; Bayley Scales-II, Reuner et al. 2008). To achieve a comparable measurement to IQ for toddlers, the Developmental Quotient (DQ) was used and calculated as follows: DQ = developmental age/chronological age × 100. Full scale IQ is reported according to the full scale IQ of the respective instrument or the DQ. For Module 3 and 4 non-verbal IQ was calculated from subtests of HAWIK-III, HAWIK-IV, CPM/SPM, Culture Fair Intelligence Test and Snijders–Oomen nonverbal Intelligence Test; verbal IQ was taken from HAWIK-III, HAWIK-IV Peabody Picture Vocabulary Test. Verbal and non-verbal IQ data for Module 1 and Module 2 were only available for < 20% of the patients and are therefore not reported.

All parents and adult patients additionally participated in a semi-structured medical history interview to determine pre- and perinatal as well as psycho-social risk factors and socio-economic status.

Best estimate clinical diagnosis (BEC) of ASD according to ICD-10 was established after obtaining the medical history, a medical exam, ADOS, ADI-R, and additional parent and teacher rating scales. The diagnostic process in total lasted 6–8 h. The ADOS module was selected based on the eligibility criteria as described in the manual and lasted 1–1.5 h. The diagnostic groups assessed were autism (F84.0), autism spectrum (F84.1, F84.5, excluding autism patients) and non-ASD. A non-ASD psychiatric diagnosis was established according to ICD-10 (World Health Organization 1992) based on expert clinical judgment and additional information from disorder specific reports by parents, teachers, and patients, such as the Child Behavior Checklist, the Youth Self Report, Teacher Report Form (Döpfner et al. 2014) or scales from the DISYPS- II (Döpfner et al. 2008).

### Statistical Analysis

Due to deviation from normal distribution, we compared group differences regarding age and IQ between diagnostic subgroups (autism, autism-spectrum, non-ASD diagnosis) by Kruskal–Wallis tests. Gender distribution was compared by χ^2^-test. Diagnostic validity was assessed by receiver operating curves (ROC). ROC is widely used to evaluate diagnostic tests with a dichotomous outcome. ROC represents the plot of sensitivities (true positives) versus specificities (1-true negatives), the resulting area under the curve (AUC) measures test accuracy. An AUC of 1 represents perfect classification, 0.5 a random result. AUC, sensitivities, and specificities with 95% confidence intervals (CI) were calculated according to published ADOS and ADOS-2 algorithm rules and cut-offs. We implemented the combined SA and RRB scores for the ADOS-2 (Lord et al. 2000; Lord and Rutter 2012; Hus and Lord 2014). We used logistic regression to study the diagnostic validity of the ADOS-2 algorithm score in combination with the SCQ and SRS in Modules 3 and 4. We compared four different models per module with subsequent integration of the two predictor variables (SCQ, SRS), separately and combined, including the ADOS-2 algorithm alone. The best fitting model was selected according to change in likelihood ratio statistics comparing each model to the baseline (ADOS-2 algorithm). We used chi-squared Wald statistics to assess the contribution of the predictors to the model. IBM SPSS statistics versions 23 and 24 were used for all statistical analyses.

## Results

### Sample Characteristics

We examined *N* = 826 individuals; *n* = 156 by Module 1, *n* = 151 by Module 2, *n* = 372 by Module 3 and *n* = 147 by Module 4. Age at time of diagnosis ranged from 2 to 40 years. Across all modules, > 80% of the participants were male. For participants with (obtained) IQ data, IQ total scores ranged from 15 to 147. Detailed information on the sample is given in Table 1. We classified diagnoses other than ASD in other developmental disorders, externalizing and internalizing disorders. Externalizing disorders included attention deficit disorders with hyperactivity (F90), attention deficit disorders (F98.8), conduct disorders (F91) and transient tic disorders (F95.0). Internalizing disorders included depressive disorders (F32), emotional disorders with onset specific to childhood (F93), obsessive compulsive disorders (F42), social phobias (F40.1), elective mutism (F94.0) and adjustment disorders (F43.2).

**Table 1:** Sample description: gender, age, IQ

		Autism (*n* = 455)	Autism spectrum (*n* = 216)	Non-ASD (*n* = 155)	Statistical group differences	
					*F*/*x*^*2*^*(df)*	*p*
Module 1 (*n* = 156)	Age *M (SD)* *n*	5.15 (1.81) 123	5.86 (2.26) 11	5.38 (1.81) 22	2.079 (2)	0.354
	FSIQ *M (SD)* *n*	62.38 (24.16) 52	87.71 (32.94) 7	65.56 (27.07) 5	3.916 (2)	0.141
	Female *n* (%)	27 (21.9)	4 (36.4)	3 (13.6)	2.231 (2)	0.328
Module 2 (*n* = 151)	Age *M (SD)* *n*	6.60 (1.86) 78	6.13 (1.63) 40	6.16 (1.56) 33	2.634 (2)	0.267
	FSIQ *M (SD)* *n*	82.96 (21.11) 57	98.57 (21.39) 28	90.86 (19.97) 21	8.232 (2)	0.016
	Female *n* (%)	19 (24.4)	7 (17.5)	6 (18.2)	0.974 (2)	0.614
Module 3 (*n* = 372)	Age *M (SD)* *n*	10.25 (2.18) 170	10.38 (2.24) 120	9.99 (2.24) 82	2.302 (2)	0.316
	FSIQ *M (SD)* *n*	94.99 (19.05) 142	102.30 (17.06) 81	98.12 (18.09) 49	10.635 (2)	0.005
	VIQ *M (SD)* *n*	99.28 (21.57) 87	103.52 (18.32) 64	102.83 (19.70) 36	2.485 (2)	0.289
	NVIQ *M (SD)* *n*	97.60 (17.32) 136	105.58 (15.75) 81	99.96 (18.61) 46	12.636 (2)	0.002
	Female *n* (%)	18 (10.6)	9 (7.5)	16 (19.5)	7.164	0.028
Module 4 (*n* = 147)	Age *M (SD)* *n*	17.98 (4.75) 84	18.40 (5.05) 45	16.31 (3.52) 18	4.090 (2)	0.129
	FSIQ *M (SD)* *n*	97.40 (20.00) 77	96.59 (19.73) 34	98.23 (17.02) 13	0.076 (2)	0.962
	VIQ *M (SD)* *n*	101.72 (24.55) 39	101.00 (25.47) 27	101.18 (17.76) 11	0.067 (2)	0.967
	NVIQ *M (SD)* *n*	99.88 (18.87) 61	94.18 (23.13) 30	99.63 (16.16) 11	0.963 (2)	0.618
	Female *n* (%)	10 (11.9)	12 (26.6)	7 (38.8)	8.788	0.012

Age reported in years, IQ reported in standard scores (100 ± 15), *FSIQ* Full Scale IQ, *VIQ* verbal IQ, *NVIQ* non-verbal IQ

In Module 1, most children (*n* = 123, 78.8%) were diagnosed with autism, *n* = 11 (7.1%) were classified as autism spectrum patients. *N* = 22 (14.1%) children were included in the non-ASD group. Other developmental disorders were diagnosed in *n* = 18 (11.7%) children; two children were diagnosed with an externalizing psychiatric disorder, one with an internalizing psychiatric disorder; one child did not show a psychiatric or developmental disorder. The male to female ratio was 3.5:1 in the autism group, 1.75:1 in the autism spectrum group and 6.3:1 in the non-ASD group. Mean IQ was 65.39 (*SD* = 26.34) in the autism and autism spectrum group combined, and 65.56 (*SD* = 27.07) in the non-ASD group. *N* = 37 (23.7%) of the participants in Module 1 were without speech. Age ranged from 2 to 12 years. Mean age was 5.21 years (*SD* = 1.85) in the autism and autism spectrum group combined, and 5.38 years (*SD* = 1.81) in the non-ASD group.

In Module 2, *n* = 78 (51.7%) of the children were diagnosed with autism and *n* = 40 (26.5%) with autism spectrum. A total of *n* = 33 (21.8%) were included in the non-ASD group. In this group, *n* = 14 children were diagnosed with other developmental disorders, *n* = 12 children with an externalizing disorder, *n* = 3 with an internalizing disorder, and *n* = 4 children received no diagnosis. The male to female ratio was 3.1:1 in the autism group, 4.7:1 in the autism spectrum group and 4.5:1 in the non-ASD group. Mean IQ was 88.11 (*SD* = 22.33) in the autism and autism spectrum group combined, and 90.86 (*SD* = 19.97) in the non-ASD group. In Module 2, age ranged from 3 to 12 years. Mean age was 6.44 years (*SD* = 1.79) for the combined autism and autism spectrum group, and 6.16 years (*SD* = 1.56) in the non-ASD group.

In Module 3, *n* = 170 (45.7%) of the participants received an autism diagnosis and *n* = 120 (32.3%) an autism spectrum diagnosis. A total of *n* = 82 (22.0%) children were included in the non-ASD group. In this group, *n* = 12 children were diagnosed with other developmental disorders, *n* = 46 children with externalizing disorders, *n* = 21 participants were diagnosed with internalizing disorders, and *n* = 3 did not show a psychiatric or developmental disorder. The male to female ratio was 8.5:1 in the autism group, 12.3:1 in the autism spectrum group, and 4.2:1 in the non-ASD group. Mean IQ was 97.54 (*SD* = 18.55) in the autism and autism spectrum group combined, and 98.12 (*SD* = 18.09) in the non-ASD group. Age in Module 3 ranged from 5 to 16 years. Mean age was 10.30 years (*SD* = 2.20) in the autism and autism spectrum group, and 9.99 years (*SD* = 2.24) in the non-ASD group.

Of the participants in Module 4, *n* = 84 (57.2%) were diagnosed with autism and *n* = 45 (30.6%) with autism spectrum. *N* = 18 (12.2%) participants were included in the non-ASD group. Of those, *n* = 3 participants were diagnosed with another developmental disorder, *n* = 5 participants with an externalizing disorder, *n* = 6 participants were diagnosed with an internalizing disorder and *n* = 4 received no diagnosis. The male to female ratio was 7.4:1 in the autism group, 2.7:1 in the autism spectrum group, and 1.5:1 in the non-ASD group. Mean IQ was 97.15 (*SD* = 19.84) in the autism and autism spectrum group combined, and 98.23 (*SD* = 17.02) in the non-ASD group. Age in Module 4 ranged from 12 to 40 years, with a mean age of 18.13 years (*SD* = 4.84) in the autism and autism spectrum group combined and 16.31 years (*SD* = 3.52) in the non-ASD group.

Comparison of diagnostic subgroups regarding age showed no group differences across all modules. In Module 2 and 3, a significant difference in full scale IQ levels and non-verbal IQ levels (Module 3) was shown with higher IQ in the non-ASD and autism spectrum group compared to the autism participants. No significant difference in verbal IQ across diagnostic groups could be shown in Module 3 and 4. Gender differences between groups were found for Modules 3 and 4 (see Table 1).

### Diagnostic Validity

We compared algorithm scores for the original algorithm and ADOS-2 algorithm against clinical diagnoses (see Table 2). An overview of ROC-AUC results and CIs are given for each module. AUCs in this sample ranged from 0.34 to 1.00. AUC > 0.80 indicates good diagnostic accuracy. We observed these values for most diagnostic comparisons (see Table 2). AUC < 0.80 was found for Module 1 ADOS and ADOS-2 algorithms differentiating non-ASD from autism, autism spectrum or the combined autism and autism spectrum group as well as for Module 4 ADOS and ADOS-2 algorithm scores differentiating autism spectrum and the combined autism and autism spectrum group from non-ASD. CIs (95%) of ADOS and ADOS-2 AUCs overlapped for all modules and diagnostic comparisons except in the case of the ADOS-2 Module 2 group younger than 5 years algorithm, which showed an AUC = 1.0 in a very small sample (*n* = 12 vs. *n* = 4).

**Table 2:** Comparison of receiver operating characteristics for published ADOS and ADOS-2 cut-offs

			Autism versus non-ASD		Autism spectrum versus non-ASD		Autism versus autism spectrum		Autism and autism spectrum versus non-ASD	
			AUC [95% CI]	Sens/Spec	AUC [95% CI]	Sens/Spec	AUC [95% CI]	Sens/Spec	AUC [95% CI]	Sens/Spec
Module 1	Total score ADOS		0.66 [0.50, 0.82]	0.93/0.50	0.37 [0.17, 0.56]	0.63/0.23	0.89 [0.74, 1.00]	0.94/0.82	0.64 [0.48, 0.79]	0.96/0.23
	*n*		123/22		11/22		123/22		134/22	
	Total score ADOS-2	No words	0.57 [0.26, 0.88]	0.83/0.20	0.40 [0.00, 0.84]	1.00/0.20	0.62 [0.13, 1.00]	0.83/0.67	0.56 [0.25, 0.86]	1.00/0.20
		*n*	29/5		3/5		29/3		32/5	
		Some words	0.69 [0.50, 0.87]	0.98/0.48	0.34 [0.13, 0.55]	1.00/0.18	0.97 [0.93, 1.00]	0.98/0.86	0.66 [0.48, 0.84]	0.99/0.18
		*n*	94/17		8/17		94/8		102/17	
Module 2	Total score ADOS		0.95 [0.91, 0.99]	0.78/0.94	0.82 [0.72, 0.92]	0.85/0.73	0.84 [0.76, 0.91]	0.78/0.78	0.91 [0.85, 0.96]	0.92/0.73
	*n*		78/33		40/33		78/40		118/33	
	Total score ADOS-2	Younger 5	1.00 [1.00, 1.00]	1.00/1.00	1.00 [1.00, 1.00]	1.00/1.00	0.83 [0.57, 1.00]	1.00/0.40	1.00 [1.00, 1.00]	1.00/1.00
		*n*	7/4		5/4		7/4		12/4	
		5 and older	0.92 [0.86, 0.98]	0.91/0.79	0.82 [0.70, 0.94]	0.80/0.66	0.77 [0.68, 0.86]	0.92/0.26	0.88 [0.81, 0.95]	0.91/0.66
		*n*	80/29		35/29		71/36		106/29	
Module 3	Total score ADOS		0.92 [0.88, 0.96]	0.79/0.90	0.82 [0.76, 0.89]	0.88/0.68	0.78 [0.72, 0.83]	0.79/0.69	0.88 [0.83, 0.93]	0.93/0.68
	*n*		170/82		120/82		170/120		290/82	
	Total score ADOS-2		0.91 [0.87, 0.95]	0.88/0.82	0.81 [0.75, 0.88]	0.88/0.66	0.75 [0.69, 0.80]	0.88/0.56	0.87 [0.82, 0.91]	0.91/0.66
	*n*		170/82		120/82		170/120		290/82	
Module 4	Total score ADOS		0.93 [0.88, 0.98]	0.70/1.00	0.77 [0.63, 0.91]	0.91/0.61	0.77 [0.69, 0.86]	0.70/0.82	0.87 [0.80, 0.95]	0.95/0.61
	*n*		84/18		45/18		84/45		129/18	
	Total score ADOS-2		0.89 [0.82, 0.96]	0.89/0.67	0.71 [0.57, 0.85]	0.84/0.28	0.74 [0.65, 0.84]	0.89/0.29	0.83 [0.74, 0.91]	0.93/0.29
	*n*		84/18		45/18		84/45		129/18	

*AUC* area under the curve, *CI* confidence interval

Sensitivity of the ADOS-2 algorithm was slightly higher than the ADOS algorithm for Module 1, some words, and for some comparisons of the two ADOS-2 Module 2 algorithm scores. For Module 3 and 4, ADOS-2 sensitivity slightly increased for the diagnostic differentiation of autism versus non-ASD and autism versus autism spectrum. For the other diagnostic comparisons, a small decrease was found. Specificity was especially low (< 0.30) for both, ADOS and ADOS-2, Module 1 differentiating non-ASD from autism and autism-spectrum. Also, for ADOS-2 Module 4, a low specificity < 0.30 was observed differentiating non-ASD from autism spectrum and from autism and non-autism spectrum combined (see Table 2).

Considering gender imbalance in Modules 3 and 4 ROCs were also calculated for male patients only. Results remained nearly identical to the combined female and male sample.

### Testing for Improved Classification by Adding Information from the SCQ and the SRS

Datasets with complete ADOS-2, SRS and SCQ data were used to determine the benefit of adding screening questionnaires to the diagnostic process for Modules 3 and 4. We entered dichotomized SRS and SCQ together with ADOS-2 into a logistic binary regression to examine possible increase of correct classifications of autism spectrum and autism as well as autism spectrum and autism combined compared to only using ADOS-2 data. For every diagnostic group 4 models were build: In model 1 only ADOS-2 data were entered, model 2 was built with ADOS-2 and SRS data, in model 3 ADOS-2 and SCQ data were entered, in model 4 we examined ADOS-2 data in combination with data from SRS and SCQ.

For ADOS-2 Module 3, classifying autism and autism spectrum combined from non-ASD model 4 with inclusion of all predictors showed the best accuracy (χ^2^ = 93.36, *p* < 0.000, − 2 Log Likelihood = 160.87) and explanatory value (Nagelkerkes R^2^ = 0.49). Still, the rate of correctly classified participants did not improve compared to the other models. The model did not show a significant improvement compared to the model including only ADOS-2 (see Table 3).

**Table 3:** Module 3: Classification by ADOS-2, SCQ and SRS

	Model fit			Correct classification	Variables	*b* (SE)	Wald (*df* = 3.84)	Odd`s ratio	*p*
	χ^2^	− 2 LL	R^2^	(%)					
Autism and autism spectrum classification (*n* = 247)									
Model 1	73.09 (*p* < 0.000)	181.15	0.40	86.2	ADOS-2	3.07 (0.39)	62.1	21.56	0.000
Model 2	89.03 (*p* < 0.000)	165.21	0.47	86.2	ADOS-2 SRS	3.04 (1.60) 1.60 (0.41)	52.83 15.09	20.91 4.94	0.000 0.000
Model 3	85.58 (*p* < 0.000)	168.65	0.46	86.2	ADOS-2 SCQ	3.16 (0.42) 1.54 (0.44)	57.07 12.21	23.70 4.69	0.000 0.000
Model 4	93.36 (*p* < 0.000)	160.87	0.49	85.4	ADOS-2 SRS SCQ	3.11 (0.43) 1.23 (0.45) 1.02 (0.49)	51.78 7.60 4.35	22.44 3.44 2.77	0.000 0.006 0.037
Autism classification (*n* = 165)									
Model 1	78.47 (*p* < 0.000)	127.17	0.53	86.1	ADOS-2	3.47 (0.46)	57.31	32.31	0.000
Model 2	92.31 (*p* < 0.000)	113.33	0.60	86.1	ADOS-2 SRS	3.39 (0.50) 1.81 (0.51)	46.53 12.76	29.85 6.13	0.000 0.000
Model 3	98.18 (*p* < 0.000)	107.46	0.63	86.1	ADOS-2 SCQ	3.89 (0.59) 2.27 (0.59)	44.36 15.03	48.83 9.68	0.000 0.000
Model 4	102.03 (*p* < 0.000)	103.61	0.55	86.1	ADOS-2 SRS SCQ	3.76 (0.53) 1.09 (0.56) 1.83 (0.63)	40.67 3.75 8.40	43.14 2.97 6.21	0.000 0.053 0.004
Autism spectrum classification (*n* = 134)									
Model 1	44.51 (*p* < 0.000)	134.48	0.38	79.9	ADOS-2	2.69 (0.45)	36.12	14.82	0.000
Model 2	53.99 (*p* < 0.000)	124.99	0.45	79.9	ADOS-2 SRS	2.74 (0.47) 1.37 (0.46)	32.95 8.89	15.47 3.92	0.000 0.003
Model 3	50.24 (*p* < 0.000)	128.75	0.42	79.9	ADOS-2 SCQ	2.82 (0.47) 1.12 (0.47)	35.54 5.52	16.79 3.05	0.000 0.019
Model 4	55.50 (*p* < 0.000)	123.50	0.46	79.9	ADOS-2 SRS SCQ	2.81 (0.49) 1.12 (0.50) 0.65 (0.53)	32.98 5.07 1.51	16.63 3.07 1.92	0.000 0.024 0.220

*L* log likelihood, *R2* Nagelkerkes R2, *b* regression coefficient, *SE* standard error, *SRS* Social Responsiveness scale, *SCQ* Social Communication Questionnaire

Model accuracy (χ^2^ = 102.03, *p* < 0.000, − 2 Log Likelihood = 103.61) for autism versus non-ASD was again best for model 4 including all predictors, explanatory value (Nagelkerkes R^2^ = 0.63) was highest for model 3 (including only ADOS-2 and SCQ) (see Table 3). Still, the rate of correctly classified participants did not improve compared to the other models. Both models did not show a significant improvement compared to the model including only ADOS-2 (see Table 3).

For the regression model comparing autism spectrum with non-ASD diagnoses in Module 3 model 4 with inclusion of all predictors showed the best model accuracy (χ^2^ = 55.50, *p* < 0.000, − 2 Log Likelihood = 123.50) and explanatory value (Nagelkerkes R^2^ = 0.46). However, the rate of correctly classified participants did not improve compared to the other models, and the model did not show a significant improvement compared to the model including only ADOS-2 (see Table 3).

Overall the number of correctly classified participants in Module 3 did not increase when adding the dichotomized questionnaires in the binary regression analysis, and the models with the questionnaires did not show a significant improvement compared to the model including only ADOS-2.

Classifying autism and autism spectrum combined from non-ASD by ADOS-2, Module 4, the first model including only ADOS-2 showed best model accuracy (χ^2^ = 3.37, *p* < 0.066, -2 Log Likelihood = 51.67). Correct classification remained constant when adding SCQ and SRS data (see Table 4).

**Table 4:** Module 4: Classification by ADOS-2, SCQ and SRS

	Model fit			Correct classification	Variables	*b* (SE)	Wald (*df* = 3.84)	Odd`s ratio	*p*
	χ^2^	− 2 LL	*R* ^*2*^	(%)					
Autism and autism spectrum classification (*n* = 175)									
Model 1	3.37 (*p* = 0.066)	51.67	0.08	88.0	ADOS-2	1.61 (0.83)	3.79	5.00	0.052
Model 2	3.49 (*p* = 0.174)	51.54	0.09	88.0	ADOS-2 SRS	1.59 (0.83) – 0.38 (1.13)	3.70 0.68	4.9 0.68	0.054 0.734
Model 3	4.12 (*p* = 0.127)	50.92	0.10	88.0	ADOS-2 SCQ	1.39 (0.87) 0.69 (0.78)	2.55 0.78	4.00 2.00	0.110 0.376
Model 4	4.32 (*p* = 0.229)	50.72	0.11	88.0	ADOS-2 SRS SCQ	1.33 (0.88) – 0.48 (1.13) 0.74 (0.79)	2.29 0.18 0.86	3.73 0.62 2.09	0.132 0.671 0.354
Autism classification (*n* = 55)									
Model 1	10.62 (*p* = 0.001)	38.40	0.30	83.6	ADOS-2	2.59 (0.83)	9.70	13.33	0.002
Model 2	10.64 (*p* = 0.005)	38.38	0.30	85.5	ADOS-2 SRS	2.59 (0.83) 0.14 (1.30)	9.64 0.12	13.45 1.15	0.002 0.914
Model 3	13.75 (*p* = 0.001)	35.27	0.37	87.3	ADOS-2 SCQ	2.85 (0.93) 1.57 (0.93)	9.42 2.85	17.33 4.82	0.002 0.091
Model 4	14.04 (*p* = 0.003)	50.72	0.38	87.3	ADOS-2 SRS SCQ	2.85 (0.94) – 0.77 (1.47) 1.75 (0.99)	9.25 0.27 3.97	17.28 0.46 5.75	0.002 0.604 0.046
Autism spectrum classification (*n* = 29)									
Model 1	0.58 (*p* = 0.446)	35.34	0.03	69.0	ADOS-2	0.69 (0.90)	0.59	2.00	0.442
Model 2	1.28 (*p* = 0.528)	34.64	0.06	69.0	ADOS-2 SRS	0.64 (0.91) – 0.93 (1.18)	0.49 0.61	1.89 0.39	0.484 0.434
Model 3	1.28 (*p* = 0.528)	34.64	0.06	69.0	ADOS-2 SCQ	0.44 (0.96) 0.75 (0.89)	0.22 0.70	1.56 2.11	0.642 0.402
Model 4	2.05 (*p* = 0.562)	33.87	0.09	69.0	ADOS-2 SRS SCQ	0.33 (1.01) – 0.99 (1.21) 0.81 (0.92)	0.11 0.67 0.77	1.39 0.37 2.26	0.740 0.412 0.379

*L* log likelihood, R. Nagelkerkes R2, *b* regression coefficient, *SE* standard error, *SRS* Social Responsiveness scale, *SCQ* Social Communication Questionnaire

In the logistic regression classifying autism versus non-ASD the rate of correctly classified participants increased (87.3% vs. 83.6%) when SRS and SCQ data were added. The best fitting model included ADOS-2, SRS and SCQ (χ^2^ = 14.04, *p* < 0.003, − 2 Log Likelihood = 50.72, Nagelkerkes R^2^ = 0.38). This model showed a significant improvement concerning the right classification rate compared to the models including only ADOS-2, or ADOS-2 and SRS (see Table 4).

Differentiating autism spectrum from non-ASD the rate of correctly classified participants (69%) and model fit did not improve when SRS and SCQ data were added (see Table 4).

## Discussion

In this study, we compared the diagnostic accuracy of the original ADOS and the new ADOS-2 algorithms to differentiate autism and autism spectrum versus non-ASD in a large German sample. For Modules 3 and 4 sample size was large. Modules 1 and 2 were also studied, but due to necessary differentiation into subgroups for the ADOS-2 algorithm, small sample sizes resulted in broad CIs and an increased risk of biased results due to especially small non-ASD samples. We were able to confirm, in accordance with the findings in the original US-American studies, that there are no differences in AUC between ADOS and ADOS-2 algorithms (Gotham et al. 2007, 2008; Bildt et al. 2009; Kamp-Becker et al. 2013; Hus and Lord 2014; Oosterling et al. 2010). The ADOS-2 Module 2 group younger than 5 years algorithm showed higher AUC than the original ADOS Module 2 algorithm, but in a very small sample, so these results must be viewed with caution.

All diagnostic ADOS and ADOS-2 algorithms except for Module 1 showed a satisfactory AUC > 0.80 for diagnosing autism and non-autism spectrum combined versus non-ASD. This diagnostic classification reflects the current DSM-5 criteria. Sample characteristics, especially of clinical samples including young children with any kind of developmental and psychiatric disorder, may be a reason for the lower AUC in Module 1. The original ADOS norming sample in Germany exhibited a satisfactory sensitivity of 90.4% and low specificity of 48.1% (Bölte and Poustka 2004). Studies on screening instruments in young children with ASD, such as studies on the M-CHAT or the revised M-CHAT have also resulted in rather low AUC-values due to low specificity (AWMF 2016, awmf.org). This reflects the complex differential diagnostic assessment in young children with suspected ASD, and the difficulty differentiating children with ASD by behavioral measures in this age group. Descriptively, Module 1, some words, exhibited slightly improved sensitivity, and marginally lower to similar specificity across all groups compared to the ADOS algorithm. Apart from the very small sample size, findings are limited by missing IQ data for individuals assessed by Module 1, which likely is due to too difficult test requirements for the young and strongly affected children.

Similar to the samples included in studies on the ADOS-2 algorithms (Gotham et al. 2007) and earlier European replications (Gotham et al. 2008; Bildt et al. 2009; Oosterling et al. 2010) we found a descriptive increase in sensitivity and decrease in specificity when using ADOS-2 algorithms Module 2, 5 years or older for autism versus non-ASD. Differentiating autism spectrum versus non-ASD resulted in a slightly lower sensitivity and specificity of the ADOS-2 compared to the original ADOS. This replicates results from another European study (Bildt et al. 2009). A possible explanation is that children diagnosed with autism spectrum disorder do not score high in the RRB section of the ADOS-2, and thus were more accurately classified with the original ADOS. In the American original publication (Gotham et al. 2007), an increase in specificity for this algorithm was shown in a patient group with lower nonverbal and verbal IQ and higher RRB scores compared to our sample and the sample by Bildt et al. (2009).

For Module 3, as was previously shown (Bildt et al. 2009; Gotham et al. 2007; Kamp-Becker et al. 2013) use of the ADOS-2 algorithm resulted in a better-balanced sensitivity and specificity compared to the ADOS when differentiating autism from non-ASD. In a study with comparable sample characteristics (Bildt et al. 2009), diagnostic validity of the original algorithm and the ADOS-2 in differentiating autism from non-ASD was similar in terms of sensitivity, but a higher specificity of the ADOS-2 algorithm was observed in our study. When differentiating autism spectrum from non-ASD, lower specificity was observed in the ADOS-2 compared to the ADOS. These results again replicate previous findings (Gotham et al. 2007; Bildt et al. 2009). A possible explanation is that some patients score lower in ADOS-2 due to the additional RRB cut-off, because repetitive behavior may not have been present during the time of the ADOS-assessment. A German study of Module 3 participants showed the same trend of higher sensitivity and lower specificity when differentiating autism and autism spectrum cases versus non-ASD (Kamp-Becker et al. 2013). The use of additional parent- or teacher-rated questionnaire in Module 3 did not benefit the already high correct classification rate of 86.2%.

For Module 4, our study found similar AUC, with, descriptively an increase in sensitivity and reduced specificity of the ADOS-2 versus the ADOS algorithm in differentiating autism from non-ASD. Similar sensitivity and reduced specificity was shown for autism and non-autism spectrum combined versus non-ASD in ADOS-2 compared to the original ADOS. This differs from the results in the norming sample for ADOS-2 Module 4 where sensitivity for ADOS-2 was slightly lower in both algorithms but specificity was improved compared to the original algorithm (Hus and Lord 2014). A replication in a European sample of older participants (mean age between 31 and 39 years) also found a better-balanced sensitivity and specificity than our study (Bildt et al. 2016). Differences between these studies may be explained by sample characteristics. Total mean age in years of our Module 4 sample was 17.91 (*SD* = 4.7). This sample is considerably younger than the norming sample (mean age in years = 21.56, *SD* = 8.62) (Hus and Lord 2014) as well as the even older Dutch sample. In a large replication study of the ADOS-2 algorithm in Module 4 with participants without intellectual disability ADOS-2 sensitivity increased and specificity decreased similar to our results. This sample also is more similar in age (mean age in years = 18.91, *SD* = 7.64) to our study (Pugliese et al. 2015).

Correct classification in terms of differentiating autism from non-ASD improved from 83.6 to 87.3% when adding the SRS and SCQ as screening instruments to the ADOS-2 Module 4 algorithm. Especially the SCQ made a significant contribution to improve correct classification. It can be argued that the SCQ, by providing information from caregivers regarding life-time behavior outside the time-frame of ADOS, adds important data which cannot be observed during the ADOS in this older sample. Given the weak specificity observed for Module 4 we recommend the use of the SCQ as a useful addition in the diagnostic process.

Limitations of the present study are mainly the low sample sizes for Modules 1 and 2. In addition, samples were not always comparable to the original norming study samples, and for some modules, IQ and gender differences were observed between diagnostic groups. All patients were referred because of a suspected ASD diagnosis. This might have influenced especially specificity of the ADOS and ADOS-2 algorithms, which might be higher in an unselected sample. In Modules 3 and 4, only complete datasets with ADOS, SCQ and SRS could be used for the regression, resulting in a reduced sample size.

In conclusion, we have replicated the high diagnostic accuracy for Modules 2 to 4 of the ADOS-2. The results show the scope and limits of the ADOS-2 in a large clinical sample, providing a better basis for deciding its clinical use. To improve the rates of correctly diagnosed individuals, ADOS-2 Module 4 may be used in combination with the SCQ and the SRS. Regarding Module 3, the addition of the two parent questionnaires did not improve correct classification rates.

There is a need for further studies with larger samples covering the entire ASD spectrum, especially for Modules 1 and 2, with a balanced number of participants in each diagnostic group. Also, diagnostic accuracy of all ADOS-2 modules should be tested against specific control groups, such as large groups of individuals with intellectual disability without ASD, specific language impairment, social phobia, selective mutism, or conduct disorder, which are especially hard to differentiate from ASD (Cholemkery et al. 2014a, b). An improvement of diagnostic accuracy as a result of adding parent information was only shown for Module 4 but should also be tested in larger samples with complete measures.
